# Testing GxG interactions between coinfecting microbial parasite genotypes within hosts

**DOI:** 10.3389/fgene.2014.00124

**Published:** 2014-05-14

**Authors:** Joy Bose, Rebecca D. Schulte

**Affiliations:** Department of Behavioral Biology, University of OsnabrueckOsnabrueck, Germany

**Keywords:** GxG, parasite, host, multiple infection, within-host interaction, *Caenorhabditis elegans*, *Bacillus thuringiensis*, virulence

## Abstract

Host–parasite interactions represent one of the strongest selection pressures in nature. They are often governed by genotype-specific (GxG) interactions resulting in host genotypes that differ in resistance and parasite genotypes that differ in virulence depending on the antagonist’s genotype. Another type of GxG interactions, which is often neglected but which certainly influences host–parasite interactions, are those between coinfecting parasite genotypes. Mechanistically, within-host parasite interactions may range from competition for limited host resources to cooperation for more efficient host exploitation. The exact type of interaction, i.e., whether competitive or cooperative, is known to affect life-history traits such as virulence. However, the latter has been shown for chosen genotype combinations only, not considering whether the specific genotype combination per se may influence the interaction (i.e., GxG interactions). Here, we want to test for the presence of GxG interactions between coinfections of the bacterium *Bacillus thuringiensis* infecting the nematode *Caenorhabditis elegans* by combining two non-pathogenic and five pathogenic strains in all possible ways. Furthermore, we evaluate whether the type of interaction, reflected by the direction of virulence change of multiple compared to single infections, is genotype-specific. Generally, we found no indication for GxG interactions between non-pathogenic and pathogenic bacterial strains, indicating that virulence of pathogenic strains is equally affected by both non-pathogenic strains. Specific genotype combinations, however, differ in the strength of virulence change, indicating that the interaction type between coinfecting parasite strains and thus the virulence mechanism is specific for different genotype combinations. Such interactions are expected to influence host–parasite interactions and to have strong implications for coevolution.

## INTRODUCTION

Genotype by genotype (GxG) interactions are interactions between species which depend on the species’ genotypes. Thus, the exact phenotype of the interaction depends on the genotypes that are involved (Hamilton, 1980; Thompson, 2005). Such interactions can be mutualistic like cooperation (Hoeksema and Thompson, 2007; Heath, 2010; Gorton et al., 2012) or antagonistic like parasitism (Webster and Woolhouse, 1998; Carius et al., 2001; Schmid-Hempel and Ebert, 2003; Schulenburg and Ewbank, 2004; Grech et al., 2006; Lambrechts et al., 2006, 2009; Rauch et al., 2006; Salvaudon et al., 2007; de Roode and Altizer, 2010; Luijckx et al., 2011). They are one of the fundamental requirements for coevolution, as shown for the coevolution between hosts and parasites (Hamilton et al., 1990; Agrawal and Lively, 2002).

Host–parasite GxG interactions are not static. They can be influenced by abiotic factors like temperature or nutrition (Thomas and Blanford, 2003; Lazzaro and Little, 2009; Wolinska and King, 2009; Sadd, 2011; Tollenaere and Laine, 2012; Gsell et al., 2013; Mboup et al., 2013), but also biotic factors, namely a third species. Facultative symbiotic microbes have been shown to influence or even mediate GxG interactions between parasites and hosts (Oliver et al., 2005; Tetard-Jones et al., 2007; Koch and Schmid-Hempel, 2012; Rouchet and Vorburger, 2012). Recently, Seppälä et al. (2012) revealed GxG interactions between coinfecting parasite species that are furthermore influenced by the environmental factor dose. Coinfections are likely to also influence GxG interactions between host and parasite, resulting in rather complex interactions (i.e., GxGxGxE). Next to coinfections of different parasite species, coinfections of different genotypes of the same parasite species are frequently observed in nature. GxG interactions for the latter remain to be shown.

Although knowledge on the role of GxG interactions between coinfecting parasites is scarce, the consequences of two parasites simultaneously infecting the same host (i.e., coinfections) have been studied in detail. Such coinfections may either be antagonistic, like resource competition (faster but not optimal host exploitation is selected at the within-host level) or spiteful competition (competitors are directly fought even though it is costly for the actor), or they may be mutualistic like public good cooperation (produced goods can be used by all individuals causing a fitness advantage for both the producer and the recipient; Buckling and Brockhurst, 2008; Mideo, 2009). It has furthermore been described that the host immune response may interfere with the interaction between competing parasites. It may do so by either preventing infection of one species if another is present or by facilitating its infection (Cox, 2001; Read and Taylor, 2001; Mideo, 2009). Such scenarios are likely to reveal GxGxG interactions between the host and the coinfecting parasites: depending on the host genotype, infection by certain parasite genotypes may be selectively favored over other parasite genotypes.

Studies on multiple infections usually assume that one of the potential interaction mechanisms is at work. We argue that depending on the exact parasite genotypes coinfecting a host, the mechanism might be different. Most theoretical and empirical studies on multiple infections focused on parasite virulence (e.g., Alizon et al., 2013), and virulence is expected to increase (e.g., resource competition) or decrease (e.g., spiteful competition) in multiple compared to single infections depending on the exact type of interaction. Therefore, we consider the presence of multiple infections with lower and with higher virulence compared to the mean of the corresponding single infections, according to the exact parasite genotype combination tested, as an indication for such mechanisms.

Here, we used the bacterial microparasite *Bacillus thuringiensis* (Bt) and its nematode host *Caenorhabditis elegans.* This model system has been well described (Wei et al., 2003; Schulenburg and Müller, 2004; Schulte et al., 2010). Bt causes persistent gut infections in *C. elegans* that potentially lead to host death (Borgonie et al., 1995, 1996; Schulenburg and Müller, 2004). The infection is caused by oral uptake of bacterial spores during feeding. The spores are associated with crystal toxins (Cry-toxins), which destroy intestinal cells. Cry-toxins are thought to be the prime determinant for the infection (Griffitts and Aroian, 2005), but also other virulence factors like phospholipase C, proteinases and hemolysins have been described (George and Crickmore, 2012). Once host resources are made available, the spores germinate and proliferate vegetatively until cells sporulate. Both antagonists show potential for specific interactions: Bt strains show high specificity against nematodes, including *C. elegans* (Wei et al., 2003; Schulenburg and Ewbank, 2004; Schulenburg and Müller, 2004; Schulte et al., 2010), and *C. elegans* expresses specific immune reactions toward different pathogens (Alper et al., 2007; Wong et al., 2007; Schulenburg et al., 2008).

*Bacillus thuringiensis* has the potential for within-host interaction since different strains differ in their growth rates and produce different public Cry-toxins to exploit the host (Payne, 1992; Payne et al., 1993). Bt is furthermore capable of bacteriocin production, substances harming other Bt-strains (Abriouel et al., 2011), which therefore can be classified as spiteful behavior. Thus, different strains may interact differently.

Since we were especially interested in the within-host interactions, we used the same outbred host population and all possible combinations of single and double infections of two non-pathogenic and five pathogenic Bt strains. As phenotypic proxies for virulence we measured host survival and host reproduction. If parasite genotypes do not interact, multiple infections should take an intermediate value of the two corresponding single infections. Interaction however may result in reduced or decreased virulence, depending on the exact type of interaction.

Our aims for this study were (i) to test for GxG interactions between coinfecting parasite strains, i.e., whether the virulence in a coinfection is influenced by the infecting genotypes, (ii) to elucidate whether there is a general difference between single and double infections which would indicate that most interactions between coinfecting parasites are of the same type, (iii) to test whether the exact change in virulence between single and mixed infections depends on the coinfecting genotypes, indicating that the exact interaction mechanism between coinfecting genotypes is genotype specific, and (iv) to find indication for the exact interaction mechanism for specific genotype combinations.

## MATERIALS AND METHODS

### BACTERIAL STRAINS AND NEMATODES

In total, we used seven different Bt strains. The nematocidal strains B-18243, B-18245, B-18246, B-18247, and B-18679 were provided by the Agricultural Research Service Patent Culture Collection (United States Department of Agriculture). Different Bt-strains differ in their genotype and Cry-toxin production (Payne, 1992; Payne et al., 1993; Schnepf et al., 2001), as do the strains we used here (Schulte et al., 2010, H. Schulenburg, personal communication). The other two strains were non-nematocidal, thus non-pathogenic toward nematodes, namely DSM-350 (German Collection of Microorganisms and Cell Cultures) and 407 Cry- (kindly provided by Christina Nielsen-LeRoux; Lereclus et al., 1989). Prior to the experiments, all Bt strains were cultured in large quantities and stored in aliquots at -20°C (Borgonie et al., 1995).

As nematode host we used a genetically diverse and outcrossed population of *C. elegans* to simulate natural conditions. Thus, our results are likely to be valid for *C. elegans* as a species and not only for a specific genotype. The population was originally prepared by Henrique Teotónio by consecutive crosses among 16 natural isolates (Teotónio et al., 2012). At least 13 of these isolates are genetically diverse and the outcrossed population is more diverse than natural populations (Rockman and Kruglyak, 2009; Teotónio et al., 2012). This population was adapted over 10 generations in 40 replicates to our laboratory conditions. Afterward the replicates were mixed, aliquoted and cryopreserved at -80°C (Stiernagle, 2006) for later usage. Otherwise, worms were maintained following standard procedures (Stiernagle, 2006).

### EXPERIMENTAL DESIGN AND PROTOCOL

**FIGURE 1:**
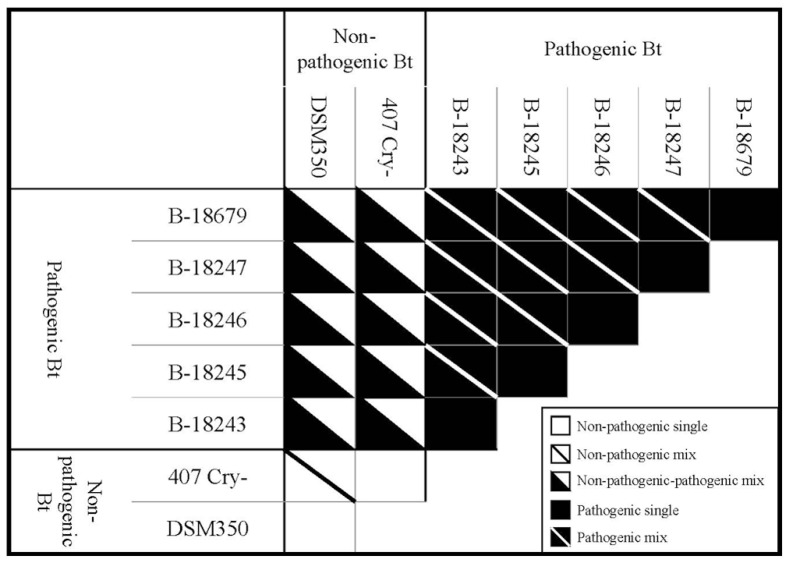
**Schematic overview of the experimental design showing different treatments and different strain combinations within each treatment.** Every strain combination was replicated four times, except for survival of the combination of 407 Cry- and B-18247 (three times).

To compare single and double infections, we tested all Bt strains in all possible combinations (**Figure 1**). Thus, we had five different treatments: non-pathogenic single Bt, non-pathogenic mixed Bt, non-pathogenic-pathogenic mixed Bt, pathogenic single Bt and pathogenic mixed Bt. In mixed infection treatments, both strains were mixed in equal proportions. Importantly, the total Bt concentration was identical in all treatments. The whole experiment was replicated four times. One replicate of the combination between 407 Cry- and B-18247 for survival was lost during experimental procedure.

The experiment was performed at 18°C and at 70% humidity. 20 age synchronized hermaphroditic worms of the last larval stage (L4) were transferred into a “worm-ball” (Sicard et al., 2007) containing peptone-free nematode-growth medium. Each worm-ball was inoculated with 3.5 × 10^7^ Bt particles and *ad libitum Escherichia coli* OP50 as a food source to prevent any effects caused by starvation. Beforehand, Bt was grown for 6 days on nematode growth medium. After 3 days, nematode survival and population size were estimated as proxies for virulence. Survival rate was measured as the number of surviving worms divided by the sum of surviving and dead worms. Population size was estimated as the number of worms per ball by washing them off with sterile water, counting twice a subsample of 20 μl and extrapolating the total number of worms. For statistical analysis of population size, we used the logarithm to the base 10.

### STATISTICAL ANALYSIS

All statistical analyses were performed using IBM SPSS Statistics Version 20.

To test for GxG interactions between coinfecting non-pathogenic and pathogenic Bt strains, we used a generalized linear model with “non-pathogenic strain,” “pathogenic strain” and the interaction term as fixed factors and survival rate and population size as dependent variables. GxG interactions are revealed by a significant interaction term. Since such a model based analysis is based on a full-factorial design, this analysis is not possible for the mixed pathogenic treatment (the five pathogenic strains cannot be grouped unequivocally into two factors which needed to be tested in all combinations; **Figure 1**).

To elucidate whether there is a general difference between single and double infections, we analyzed a general linear mixed model with “treatment” (non-pathogenic-pathogenic mixed Bt, pathogenic single Bt and pathogenic mixed Bt) as fixed factor, “combination (treatment)” as random factor and survival rate and population size as dependent variables. The exact difference between the three treatment categories was tested using LSD (Fisher’s Least Significance Difference) as *post hoc* test.

Our main question is, however, whether the change in virulence between single and multiple infections and thus the type of interaction between coinfecting strains depends on their exact genotypes. This should become visible by plotting single and multiple infections of the mixed treatments. For each mixed infection, we calculated the mean from the corresponding single infections. For this, we created random pairs of the four replicates (e.g., the mean of single infection A and B of replicate 1 was calculated). We estimated the influence of the factors multitude of infection (mean of single versus mixed infections) and strain combination (10 combinations for the non-pathogenic-pathogenic mixed and the pathogenic mixed treatment, each) and the interaction between both as fixed factors in a generalized linear model. A significant interaction term reveals in how far the difference between the mixed infection and mean single infections depended on the genotype combination tested. This analysis was performed for the mixed non-pathogenic-pathogenic and the mixed pathogenic treatments separately.

Finally, we were interested in the exact type of interaction between coinfecting strains. Depending on the interaction type, mixed infections should be more or less virulent than the mean of the corresponding single infections. Thus, we tested whether the slopes between the two infection types of each strain combination differed from zero using a 1-sample *t*-test.

## RESULTS

**FIGURE 2:**
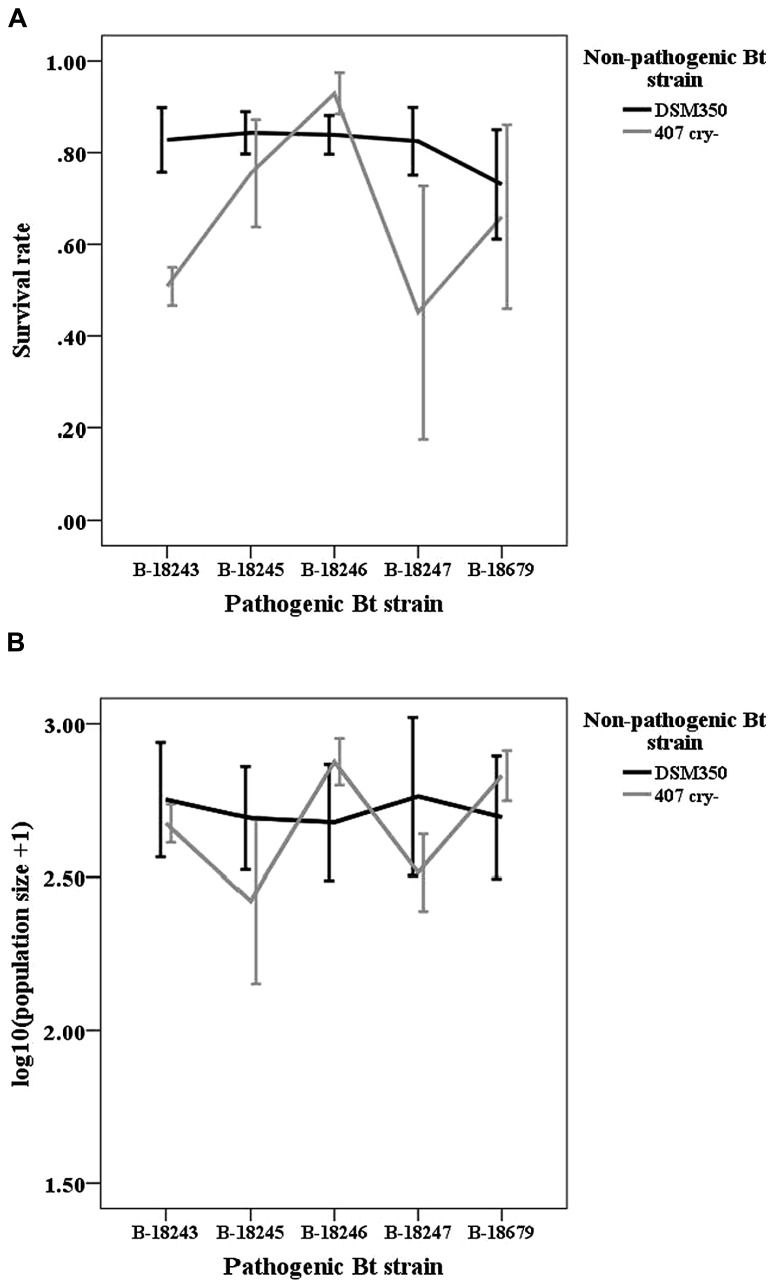
**Line graphs of (A) survival rate and (B) logarithmic population size of the non-pathogenic-pathogenic mixed treatment.** Vertical lines represent the one-fold standard error.

We did not find any indications for GxG interactions in coinfections of non-pathogenic and pathogenic strains as indicated by non-significant interaction terms (**Table 1**; **Figure 2**). However, non-pathogenic strains differed in their effect on host survival and pathogenic strains tend to differ.

**Table 1:** Statistical results of the generalized linear model testing for GxG interactions in the non-pathogenic-pathogenic mixed treatment using non-pathogenic Bt strain, pathogenic Bt strain and the interaction between both as fixed factors.

	Survival			Population size		
Treatment	Waldχ^2^	df	*p*	Waldχ^2^	df	*P*
**Non-pathogenic-pathogenic mix**	
Constant	549.780	1	**<0.001**	3095.070	1	**<0.001**
Non-pathogenic Bt	5.868	1	**0.015**	0.289	1	0.591
Pathogenic Bt	8.428	4	*0.077*	2.874	4	0.579
Non-pathogenic Bt^*^ pathogenic Bt	7.289	4	0.121	3.923	4	0.416

*Significant *p*-values are displayed in bold, trends in italics.*

**FIGURE 3:**
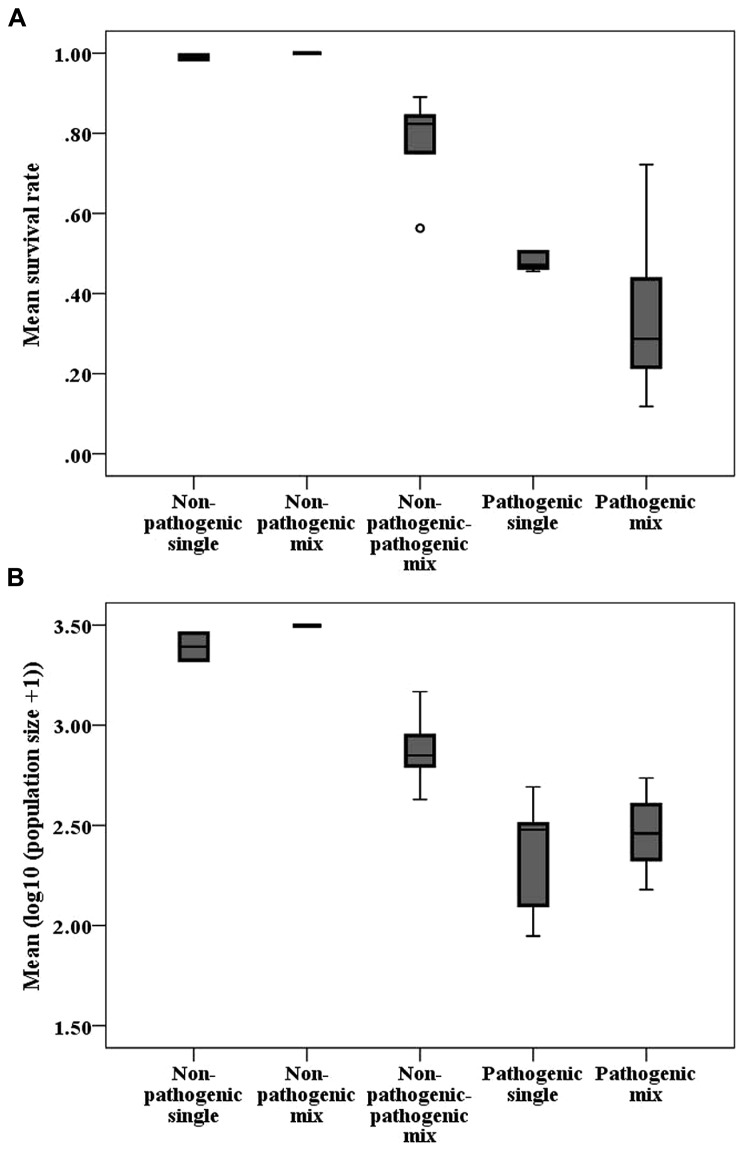
**Boxplots of (A) mean host survival rate and (B) mean logarithmic population size per strain combination of different treatments.** The horizontal line indicates the median, the box the 25% quartile above and below the median, and the whiskers the data range. Circles represent outliers and stars represent extreme values.

Generally, infection treatments differed from each other (**Table 2**; **Figure 3**). The mixed non-pathogenic-pathogenic treatment differed from the pathogenic treatments for both variables, while the two pathogenic treatments marginally differed for population size, but not for survival rate.

**Table 2:** Statistical results of the comparison of the three pathogenic treatments (mixed non-pathogenic-pathogenic Bt, pure pathogenic Bt, mixed pathogenic Bt) using a general linear mixed model with treatment as fixed factor and strain combination(treatment) as random factor and a *post hoc* test (LSD).

	Survival			Population size		
	df_n,d*_	*F*	*p*	df_n,d*_	*F*	*p*
Constant	1, 21.334	243.502	**<0.001**	1, 97.000	2252.137	**<0.001**
Treatment	2, 21.364	14.119	**<0.001**	2, 97.000	10.649	**<0.001**

***Post hoc***

Non-pathogenic-pathogenic mix – pathogenic single			**0.005**			**<0.001**
Non-pathogenic-pathogenic mix – pathogenic mix			**<0.001**			**0.003**
Pathogenic single – pathogenic mix			0.285			*0.063*

*n, numerator; d, denominator.*

*Significant results are displayed in bold, trends in italics.*

**FIGURE 4:**
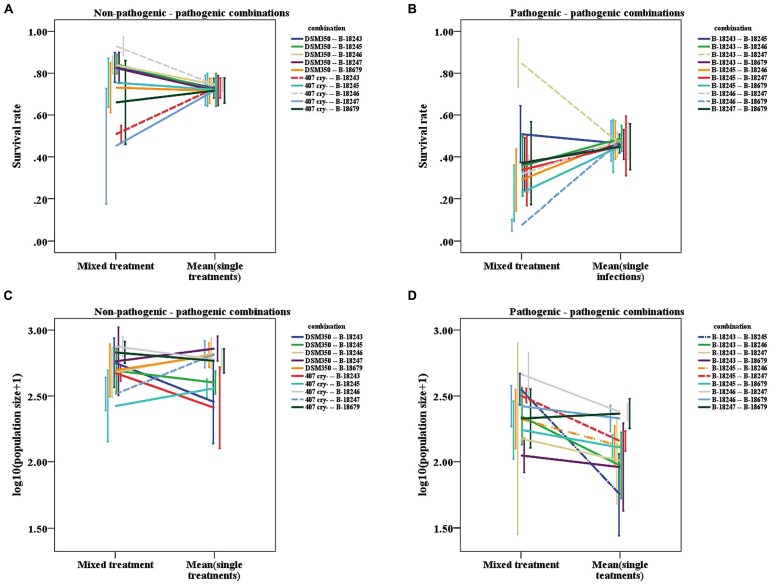
**Line graphs of mixed treatments and the mean of the corresponding two single infections. (A)** Survival rate of the non-pathogenic-pathogenic mixed treatment. **(B)** Survival rate of the pathogenic mixed treatment. **(C)** Logarithmic population size of the non-pathogenic-pathogenic mixed treatment. **(D)** Logarithmic population size of the pathogenic mixed treatment. Dashed lines represent combinations, for which the slope differs significantly from zero, dashed-dotted lines represent combinations, for which the slope shows a tendency to differ from zero. Vertical lines represent the onefold standard error.

Furthermore, we found indications that the interaction mechanism depends on the coinfecting parasite genotypes (**Table 3**). In how far the mixed treatments differ from the mean of the single values for survival depends on the exact genotype combination tested, as indicated by a trend for the interaction term for the non-pathogenic-pathogenic mixed treatment (**Figure 4A**) and a significant interaction term for the pathogenic mixed treatment (**Figure 4B**). This was not the case for population size (**Figures 4C,D**).

**Table 3:** Statistical results of the generalized linear model testing for a genotype-specific difference between single and multiple infections using multitude of infection (mean of single infections versus mixed infection), strain combination and the interaction between both as fixed factors.

	Survival			Population size		
Treatment	Waldχ^2^	df	*p*	Waldχ^2^	df	*p*
**Non-pathogenic-pathogenic mix**
Constant	1721.335	1	**<0.001**	6712.417	1	**<0.001**
Multitude of infection	0.107	1	0.743	0.001	1	0.973
Strain combination	17.740	9	**0.038**	11.552	9	0.240
Multitude × strain combination	15.647	9	*0.075*	7.749	9	0.560
**Pathogenic mix**
Constant	340.076	1	**<0.001**	2132.295	1	**<0.001**
Multitude of infection	4.512	1	**0.034**	6.443	1	**0.011**
Strain combination	17.475	9	**0.042**	9.110	9	0.427
Multitude × strain combination	18.557	9	**0.029**	5.139	9	0.822

*Significant results are displayed in bold, trends in italics.*

The significant interaction terms are not only caused by deviations in one direction. We found that for survival, the slopes of the strain combinations deviate in both directions from zero. For the non-pathogenic-pathogenic mixed treatment, we found one case in which the mix is more virulent than the mean of the single infections (407 Cry- and B-18246: 1-sample *t-*test, *t* = 8.653, df = 3, *p* = 0.003; **Figure 4A**) and one combination in which it is less virulent than the mean of the two single infections (407 Cry- and B-18243: 1-sample *t-*test, *t* = -15.236, df = 3, *p* = 0.001; **Figure 4A**). For the pathogenic mixed treatment, the mix is more virulent in one combination (B-18243 and B-18247: 1-sample *t-*test, *t* = 5.644, df = 3, *p* = 0.011; **Figure 4B**) and less in two combinations (B-18246 and B-18247: 1-sample *t-*test, *t* = -9.2724, df = 3, *p* = 0.003; B-18246 and B-18679: 1-sample *t-*test, *t* = -3.342, df = 3, *p* = 0.044; **Figure 4B**). For host population size, one combination of the non-pathogenic-pathogenic mixed treatment performed worse in mixed infections (407 Cry- and B-18247: 1-sample *t-*test, *t* = -3.669, df = 3, *p* = 0.035; **Figure 4C**). One combination of the pathogenic mixed treatment did show higher virulence in the mixed compared to the single treatments (B-18245 and B-18247: 1-sample *t-*test, *t* = 4.948, df = 3, *p* = 0.016; **Figure 4D**), two showed a trend (B-18243 and B-18245: 1-sample *t-*test, *t* = 2.950, df = 3, *p* = 0.060; B-18245 and B-18246: 1-sample *t-*test, *t* = 2.759, df = 3, *p* = 0.070; **Figure 4D**).

## DISCUSSION

Theoretical models on multiple infections usually assume only one mechanism of parasite interaction to be present, although different interaction types like resource competition or public good cooperation have been described (Buckling and Brockhurst, 2008; Mideo, 2009). Furthermore, GxG interactions between coinfecting parasites occur (Seppälä et al., 2012), which may influence the parasite interaction. Here, our aim was to test whether the interaction mechanism can be determined by GxG interactions between coinfecting parasites.

We could not show that the virulence of coinfections depends on GxG interactions between both infecting strains. Yet, we cannot exclude their presence since we were only able to test for such interactions in the non-pathogenic-pathogenic mixed treatment, and not in the pathogenic mixed treatment. The latter might have been more suitable for such an analysis since both and not only one strain are pathogenic and reduce fitness. We also only tested a rather small set of non-pathogenic strains in a low sample size, which might have obscured GxG interactions.

However, we provide evidence that within-host interactions of Bt genotypes follow a general pattern: Over all tested genotype combinations, we found that mixed non-pathogenic-pathogenic combinations are less virulent than pathogenic combinations (**Figure 3**). This may be explained by the infection characteristics of this model system (Schnepf et al., 1998): Bt spores, which are taken up by the host are already associated with Cry-toxins. These proteins are of prime importance for the infection, since they cause pore formation in the gut epithelium. They are released and activated in the gut, thus representing a public good (Raymond et al., 2007). In mixed non-pathogenic-pathogenic combinations, only one strain produces toxins and therefore virulence is reduced. What is known about Cry-toxin genes is therefore in line with the theory on public good cooperation and non-cooperating cheaters (Brown et al., 2002; Buckling and Brockhurst, 2008; Alizon and Lion, 2011).

Although we were not able to show that coinfections are characterized by GxG interactions, the virulence difference between multiple and the corresponding single infections depends on the exact genotype combination that coinfects a host (**Figure 4**). For some genotype combinations, virulence is higher, for others it is lower in double infections compared to the mean single infections. This indicates that different interaction mechanisms may be involved in the Bt-*C. elegans* model system. For example, next to the production of public Cry-toxins, Bt may compete by producing bacteriocins (Abriouel et al., 2011). The exact interaction mechanisms, which are involved in this model system remain to be shown. Importantly, we show that the virulence difference between double and the corresponding single infections is characterized by GxG interactions within the host. These interactions were not revealed by the interaction term between the genotypes of coinfecting strains (see **Table 1**).

Surprisingly, we did not find similar trends for population size as we revealed for host survival. For example, population size is not affected by the strain combination tested but survival rate is (**Table 3**). Since we took our measurements on a certain day, we cannot draw any conclusions about lifetime fitness of hosts and about potential costs. However, *C. elegans* is known to produce the majority of offspring during the first days of adulthood (Huang et al., 2004). One explanation for our results might be that reproduction is not affected by the genotypes of Bt strains, and that hosts produce their offspring before they die. However, since they die earlier when they are infected by certain Bt genotype combinations but population size remains the same, reproduction could also be shifted toward early reproduction. Alternatively, this could be caused by host genotype effects. Since we used a genetically diverse worm population, it is possible that the host genotype influences the interaction between different bacterial genotypes.

Thus, host–parasite interactions can be influenced by genotype specific within-host interactions. Since host–parasite GxG interactions have been revealed for many model systems including the one we used here (Schulte et al., 2010), it is likely that genotype specific within-host interactions are influenced by the host genotype and vice versa, resulting in GxGxG interactions. A potential mechanism for GxGxG interactions could be immune mediated interaction between parasites: depending on the host genotype, the immune-system could be more effective against some parasite genotypes and thus facilitate infections by others (Cox, 2001; Read and Taylor, 2001; Mideo, 2009). The general idea that a third party can influence GxG interactions is not new. For example, host–parasite GxG interactions are known to be influenced or even mediated by the genotypes of host endosymbionts (Oliver et al., 2005; Tetard-Jones et al., 2007; Koch and Schmid-Hempel, 2012; Rouchet and Vorburger, 2012). The situation can even be complicated if the interactions are influences by environmental factors (Seppälä et al., 2012).

That host and parasite genotypes do interact is one of the key assumptions for mathematical models on host–parasite coevolution (Hamilton et al., 1990; Agrawal and Lively, 2002). Thus, changes of allele frequencies and changes of genotypes over time require GxG interactions to occur. Here, we reveal additionally genotype specific within-host interactions of different parasite genotypes. The resulting GxGxG interactions should result in even higher evolutionary rates. If the outcome of host–parasite GxG interactions depends on within-host parasite GxG interactions, the frequency of multiple infections should influence host–parasite coevolution. It has indeed been shown that the frequency of multiple infections has strong implications for host–parasite interactions (e.g., Turner and Chao, 1998, 1999), but also the initial frequency of each strain is of importance (Taylor et al., 1997; Harrison et al., 2006; Rumbaugh et al., 2009; Zwart et al., 2009). Thus, many factors influence host–parasite coevolution under natural condition, making the outcome and epidemiology even more difficult to predict (e.g., de Roode et al., 2005; Alizon and Lion, 2011). This may explain why examples for predicted evolutionary dynamics are rarely found in nature (but see Burdon and Thrall, 2000; Jokela et al., 2003; Ebert, 2008; Dupas et al., 2009; Laine, 2009). Within-host GxG interaction is another factor that should be considered in the study of host–parasite interactions.

Our study provides an intriguing experimental evidence for the potential importance of GxG interactions among distinct pathogen genotypes within their host. We reveal that GxG within-host interactions can occur between genotypes of the same species. Since genotypes may be more or less virulent in combination than the corresponding single infections, GxG within-host interactions are furthermore likely include different interaction mechanisms and to influence the interaction between host and parasites, which is also genotype specific in this model system. What remains to be shown in future experiments is whether the exact outcome of the interaction between different parasite genotypes also depends on the host genotype. Taken together, GxG interactions between coinfecting parasites may have strong implications for epidemiology and virulence evolution.

## AUTHOR CONTRIBUTIONS

Rebecca D. Schulte designed the experiment. Rebecca D. Schulte and Joy Bose acquired, analyzed and interpreted the data and wrote the manuscript.

## Conflict of Interest Statement

The authors declare that the research was conducted in the absence of any commercial or financial relationships that could be construed as a potential conflict of interest.
